# Epidemiology, transmission dynamics, risk factors, and future directions of rabies in the Arabian Peninsula using one health approach: a review

**DOI:** 10.1093/eurpub/ckae164

**Published:** 2025-01-13

**Authors:** Md Mazharul Islam, Aisha Naeem, Philip P Mshelbwala, Pronesh Dutta, Mohammad Mahmudul Hassan, Ahmed K. Elfadl, Chiori Kodama, Susu M Zughaier, Elmoubashar Farag, Devendra Bansal

**Affiliations:** Department of Animal Resources, Ministry of Municipality, Doha, Qatar; Research and Graduate Studies, QU Health, Qatar University, Doha, Qatar; Department of Primary Industries, NSW, Australia; Institute of Epidemiology, Disease Control & Research, Mohakhali, Dhaka, Bangladesh; Queensland Alliance for One Health Sciences, School of Veterinary Science, The University of Queensland, Gatton, Queensland, Australia; Faculty of Veterinary Medicine, Chattogram Veterinary and Animal Sciences University, Kulshi, Chattogram, Bangladesh; Department of Animal Resources, Ministry of Municipality, Doha, Qatar; WHO Regional Office for the Eastern Mediterranean, Cairo, Egypt; College of Medicine, QU Health, Qatar University, Doha, Qatar; Health Protection and Communicable Diseases Department, Ministry of Public Health, Doha, Qatar; Health Protection and Communicable Diseases Department, Ministry of Public Health, Doha, Qatar

## Abstract

Despite global initiatives to eliminate dog-mediated human rabies by 2030, the Arabian Peninsula faces challenges due to insufficient data. This review addresses the current rabies situation and knowledge gaps in the region and proposes One Health interventions. Employing a mixed-method approach combining scoping and systematic review, the study commenced with a Delphi discussion to identify knowledge gaps and set objectives. The literature search encompassed published articles and grey literature. The spatial and temporal distribution of rabies was analysed, alongside quantitative meta-analyses to assess prevalence. Rabies virus gene sequences from the NCBI database were examined for reservoir hosts and evolutionary patterns. The final Delphi discussion with experts focused on addressing knowledge gaps and formulating One Health interventions. The first reported human rabies case in this region occurred in Saudi Arabia in 1980. Yemen reported the highest number of cases (439), followed by Iraq (249), Saudi Arabia (91), Jordan (14), and Oman (9). Fox bites accounted for the most cases (47.4%), followed by dog (36.8%) and wild animal (15.8%) bites. The virus was detected in at least 21 animal species. Phylogenetic analysis detected a single strain with two clades, with foxes being the primary virus reservoir. However, the experts expressed scepticism about the accuracy of rabies reports in scientific literature. To achieve the 2030 goal of eliminating dog-mediated human rabies, a stepwise approach towards rabies elimination assessment is crucial in the region. Enhanced surveillance, awareness campaigns, and access to post exposure prophylaxis are essential to address the disease burden.

## Introduction

Rabies, an ancient and deadly zoonotic disease, has documented instances dating back to Babylon in the 23rd century BC [1]. It claims over 59 000 lives annually [2], predominantly affecting children in impoverished communities in Africa and Asia. As a neglected tropical disease, rabies poses a significant public health concern in the Arab world [3, 4].

The causative agent is the rabies virus (RABV), which belongs to the lyssavirus genus within the Rhabdoviridae family [1]. The lyssavirus genus comprises at least 17 species, but only RABV causes neurotrophic symptoms in all warm-blooded mammals [5]. Typically, rabies symptoms develop within one to three months after the virus enters the body, although extended incubation periods have been reported [1]. Once symptoms appear, infected individuals or animals generally succumb within two weeks. While New World bats are considered the ancestors of the virus, bats primarily act as rabies reservoirs in the American region [6]. In contrast, dogs are the main reservoir and cause over 95% of human deaths in over 150 countries, predominantly in Asia and Africa [7].

The Arabian Peninsula, comprising Bahrain, Kuwait, Oman, Qatar, Saudi Arabia (KSA), the United Arab Emirates (UAE), Yemen, and the southern parts of Iraq and Jordan, holds global significance due to its robust economy, vast oil and gas reserves, diverse population, and cultural similarities [8]. Although global health agencies, including the World Health Organization (WHO), the Food and Agriculture Organization (FAO), the World Organization for Animal Health (WOAH), and the Global Alliance for Rabies Control (GARC), aim to eliminate dog-mediated human rabies deaths by 2030 [9], significant knowledge gaps remain in this region, particularly in epidemiology, transmission dynamics, risk factors, and prevention and control strategies. These gaps hinder evidence-based policy decisions. In this review, we compile and analyse data on the rabies situation at the human—animal interface in the Arabian Peninsula and propose One Health intervention approaches towards effective prevention and control in the region, aligning with the ‘Zero by 30’ global strategic plan to eliminate dog-mediated human rabies deaths by 2030.

## Methods

The review followed a mixed-method approach, incorporating elements of both scoping and systematic review methodologies. It began with a Delphi discussion to identify knowledge gaps and define study objectives, followed by systematic data search, extraction, and analysis. The study concluded with a final Delphi discussion.

### Initial Delphi discussion

On 24 March 2022, we discussed our knowledge of rabies, focusing on potential risk factors, transmission dynamics, and prevention and control measures. We also planned the data search strategy and defined the inclusion and exclusion criteria for the manuscript. In the second meeting on 05 September 2023, we reviewed updated rabies knowledge and identified key knowledge gaps. Finally, on 19 October 2023, we finalized the core knowledge gaps to discuss with regional and international experts.

### Literature search

Following PRISMA guidelines [10], the systematic data search covered PubMed, Embase, Web of Science, and Scopus for published articles on rabies in the Arabian Peninsula, without a specific timeframe. Grey literature was explored, focusing on governmental documents from these countries and non-governmental sources such as WHO, FAO, WOAH, GARC, and ProMED-mail. Additionally, manual searches covered the reference lists of included articles, Arabic journals, citizen science documents in blog posts or social media, and newspaper articles. The EndNote citation manager and Rayyan systematic review database facilitated the systematic screening for relevant articles (Supplementary Fig. S1). Finally, we searched the NCBI GenBank database for RABV gene sequences in the Arabian Peninsula.

### Data extraction and analysis

Data extraction included country name, sampling and publication year, host and reservoir information, and details about animal bites (Supplementary Table S1). We used R software (version 4.1.2) to perform descriptive statistical analyses on the included articles. We conducted quantitative meta-analyses employing a random-effects model to estimate the pooled prevalence of rabies among suspected non-human hosts with 95% confidence intervals (CI). Studies were weighted based on the inverse of their variance. The degree of heterogeneity among studies was evaluated using the Inconsistency Index (*I*^2^) and the ‘Tau-squared’ (τ^2^) test to estimate between-study variance. All meta-analysis results were presented using forest plots. Spatial variations in rabies cases were visualized through a choropleth map created in ArcMap 10.8, and temporal distribution was calculated using Microsoft Excel 2016. The RABV evolutionary pattern was analysed using the viral sequences by the Maximum Likelihood method and Tamura-Nei model in MEGA11.

### Expert panel discussion

An expert panel was hosted on 9 November 2023 by the Ministry of Public Health, Qatar. It included seven experts from the United States Centers for Disease Control and Prevention, Erasmus Medical College of the Netherlands, WHO, and WOAH, along with the authors. The panel discussed the 2030 global goal of eliminating human deaths from dog-mediated rabies in the region, prioritizing the elimination of fox-mediated rabies, the cost-effectiveness of oral rabies vaccination (ORV) for wildlife, gaps in rabies data reporting, priority research areas for public health policy decisions, and resource sustainability through the One Health approach. Expert opinions were used to address the knowledge gaps and develop further recommendations.

## Results

The initial Delphi discussions indicated that rabies is a significant but often overlooked health concern in the region. There appears to be a lack of publicly accessible information on rabies from countries in this area, and even international organizations such as the FAO, WHO, OIE, and GARC provide relatively limited data on the disease. Following a systematic data search, we identified 41 research articles, most of which were basic research (*n* = 17, 41.46%, 95% CI: 26.71–57.80), along with case reports (*n* = 10, 24.39%, 95% CI: 12.91–40.64), reviews (*n* = 8, 19.51%, 95% CI: 9.37–35.37), conference abstracts (*n* = 2, 4.88%, 95% CI: 0.85–17.81), short communications (*n* = 3, 7.32%, 95% CI: 0.91–21.01), and a letter to the editor (*n* = 1, 2.44%, 95% CI: 0.13–14.41) (Supplementary Table S2). Additionally, six web documents [2, 9, 11–14] contributed to understanding the disease in this region.

Iraq, Jordan, Qatar, and Yemen are GARC members [9]. FAO and GARC devised the Stepwise Approach towards Rabies Elimination (SARE), a tool for planning, monitoring, and evaluating rabies control efforts [9]. However, the member countries may not have undertaken a SARE assessment, and their SARE scores are unavailable on the GARC website. According to GARC, dog rabies is endemic in Iraq, Jordan, and Yemen, with approximately 18.5% vaccination coverage. The average annual human rabies deaths are 24, 1, 0, and 148 in Iraq, Jordan, Qatar, and Yemen, respectively, with corresponding rabies control costs of $9 207 391, $1 224 152, $2 072 383, and $17 110 158. Notably, Qatar has endorsed a national framework for rabies control, although the report is not publicly available [9].

### Human rabies

**Figure 1.**
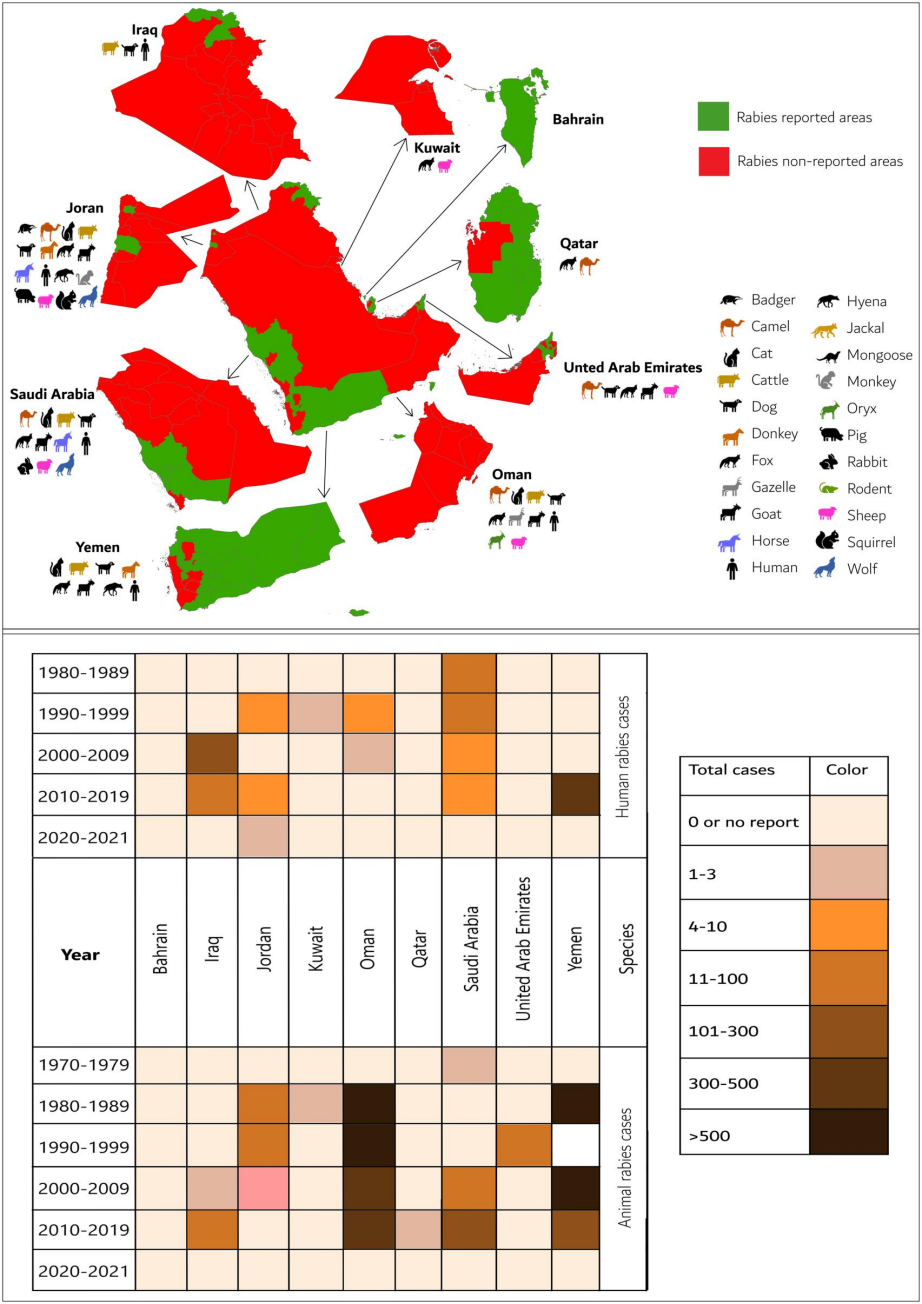
Spatial (top) and temporal (bottom) distribution of natural rabies cases at the human–animal interface in the history of Arabian Peninsula. Animal species icons in the top figure represent species previously reported in this region.

Bahrain is the only country free from both human and animal rabies. Kuwait, Qatar, and the UAE have not reported natural human rabies cases, while Yemen, KSA, and Iraq are considered endemic for human rabies (Fig. 1) [2]. Historically, Yemen has reported the highest number of human rabies cases (*n* = 439), followed by Iraq (*n* = 249), KSA (*n* = 91), Jordan (*n* = 14), and Oman (*n* = 9), with Jordan averaging two cases annually [9].

The temporal distribution of human rabies in the region began with KSA reporting the first case in 1980, followed by Oman in 1990 (Fig. 1) [3, 15]. Our analysis revealed that 47.4% (95% CI: 25.2–70.5) of the cases resulted from fox bites, followed by bites from dogs (36.8%, 95% CI: 17.2–61.4) and wild animals (15.8%, 95% CI: 4.2–40.5). Yemen, particularly among males and young individuals, exhibits high exposure to animal bites [16]. From 2007 to 2009, KSA recorded 11 069 animal bites, mainly from dogs and cats, followed by foxes and wolves [17]. Oman documented 22 778 animal bites from 1991 to 2013, primarily from foxes, followed by wolves, dogs, and wild cats, with a decline noted after 2005 [18]. Iraq reported approximately 12 358 animal bites from 2012 to 2016, predominantly in rural areas [19, 20].

Furthermore, KSA, Kuwait, Qatar, Oman, and the UAE have reported imported human rabies cases (Supplementary Fig. S2). One case each was imported to KSA from Morocco in 2016, Kuwait from India in 2015, Oman from Bangladesh in 1995, and the UAE from India in 2009 [21]. Qatar had three imported cases originating from India in 1995 [21] and Nepal in 2018 and 2019 [22]. Yemen exported one case to Russia in 1991 [23].

### Rabies in animals

**Figure 2.**
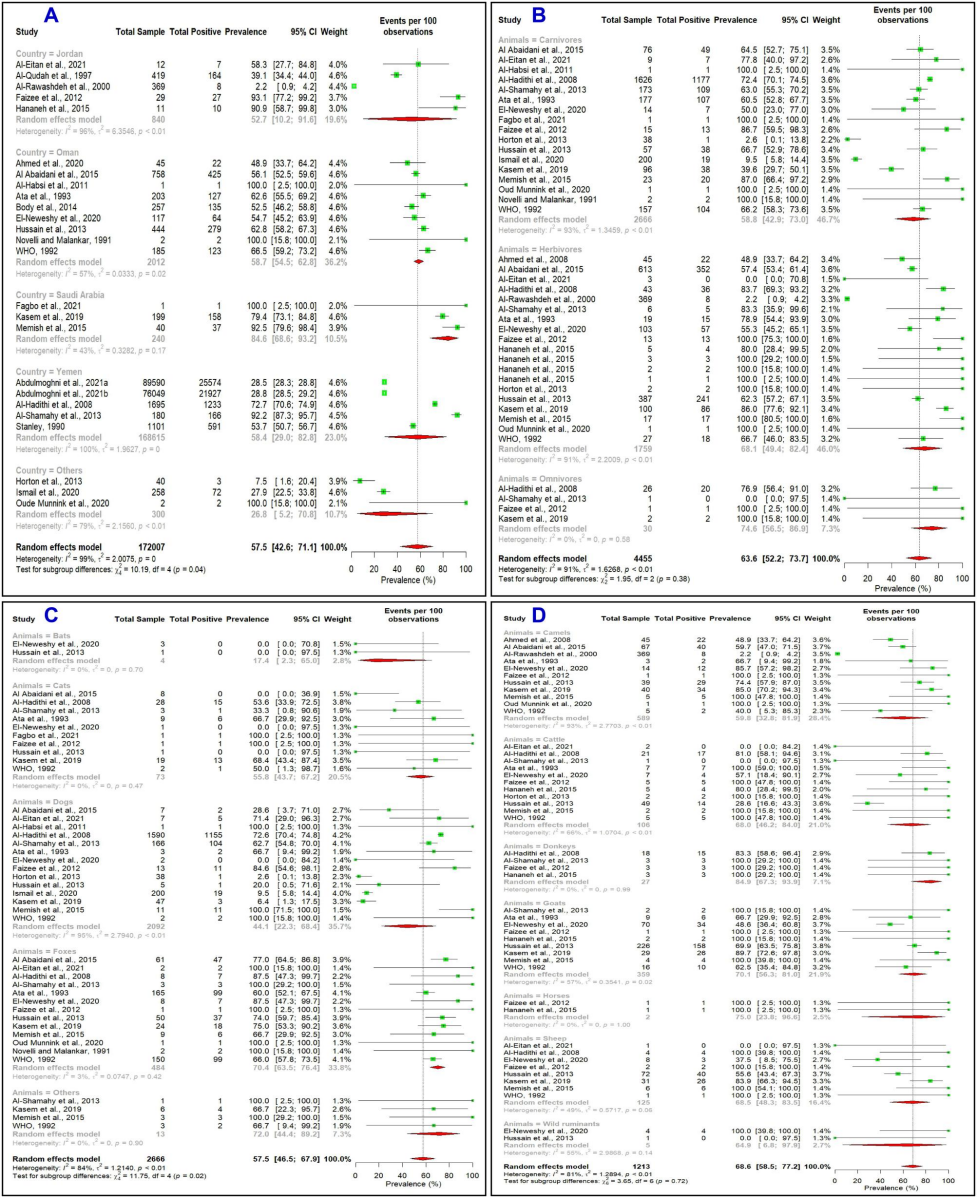
Forest plots showing the factor-wise estimated pooled prevalence of rabies among suspected non-human animals in the Arabian Peninsula. (A) Distribution of the disease by country, (B) distribution by animal type, (C) distribution among different carnivores, and (D) distribution among different herbivores.

At least 21 animal species, including carnivores, herbivores, and omnivores, have tested positive for rabies (Fig. 1). KSA showed the highest prevalence (84.6%, 95% CI: 68.6–93.2) among the suspected animals, followed by Oman (58.7%, 95% CI: 54.5–62.8), Yemen (58.4%, 95% CI: 29.0–82.8), and Jordan (52.7%, 95% CI: 10.2–91.6) (Fig. 2). Jordan is considered endemic for dog rabies [2]. Among carnivores, wild species exhibited a higher rabies prevalence. However, dogs were frequently tested, with the highest number of positive cases (*n* = 502), followed by foxes (*n* = 476) and cats (*n* = 44).

### The virus in the Arabian Peninsula

**Figure 3.**
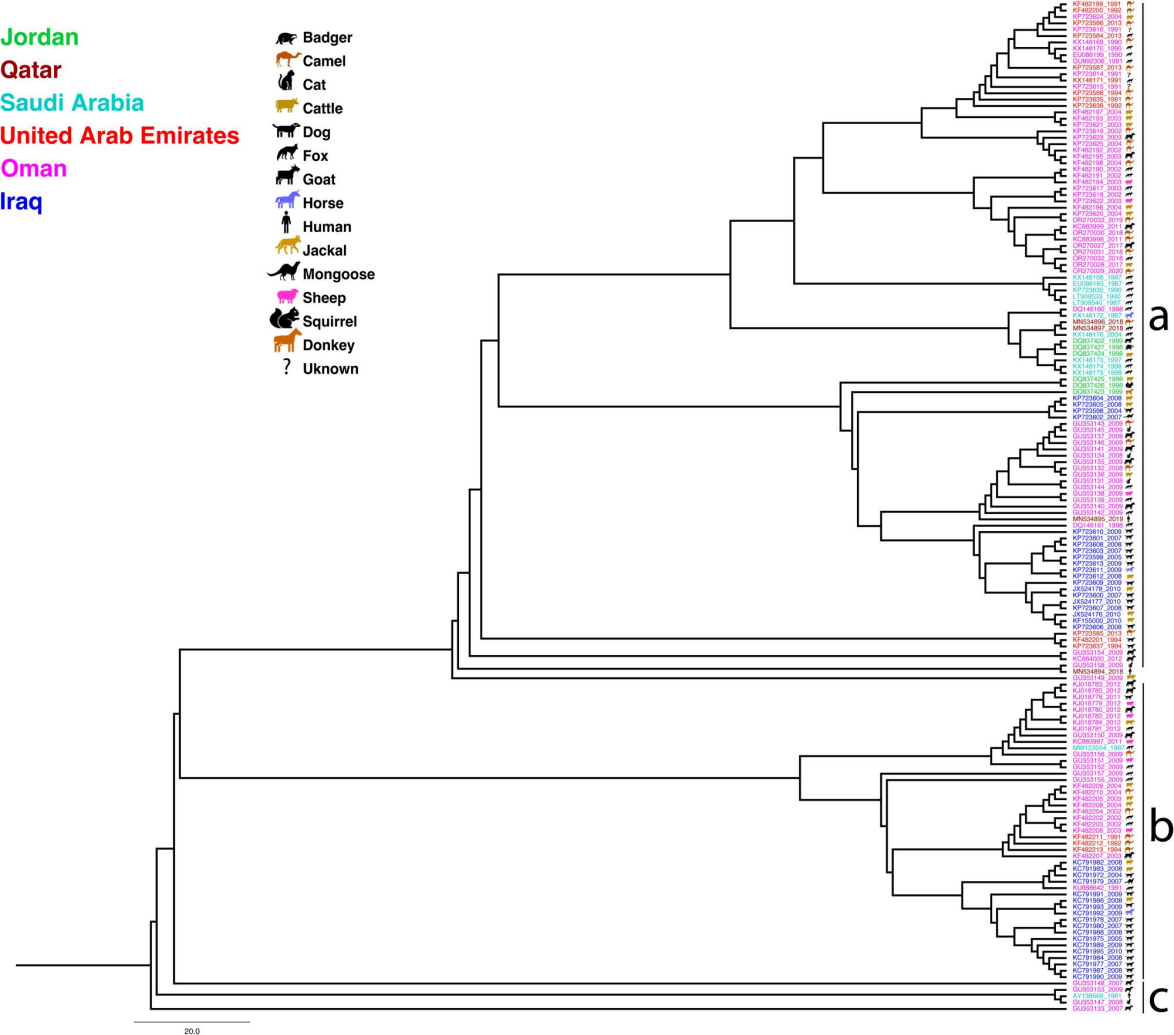
Phylogenetic tree showing the molecular relationship of the rabies virus sequences isolated from different animals in the Arabian Peninsula.

We analysed 191 RABV sequences spanning from 1987 to 2019 from Qatar, KSA, the UAE, Yemen, Oman, Iraq, and Jordan (Supplementary Table S3). Our analysis of full-length sequences revealed two prominent clades in this region: A well-supported clade ‘a’ and a smaller clade ‘b’ (Fig. 3). These clades contain strains that are not widely distributed across the region. Clade ‘a’ includes of strains from the UAE, KSA, Qatar, and Oman. The strain from Qatar [22], closely linked to strains from KSA, originated from foxes within clade “a”. Subclade ‘b’ includes strains from Iraq and Oman. Host analyses confirm that dog-derived viruses dominate in Iraq, while strains from Oman, with some introductions from the UAE, show a broad host range, including sheep, dogs, cats, cows, and camels (Fig. 3 and Supplementary Fig. S3A). Clade ‘a’ likely originated in Turkey, Israel, or Iran [24], with wolves, foxes, and dogs as predominant hosts, except for one instance in horses (KSA) and one in cattle (Israel) (Supplementary Fig. S3B).

### Possible transmission dynamics

**Figure 4.**
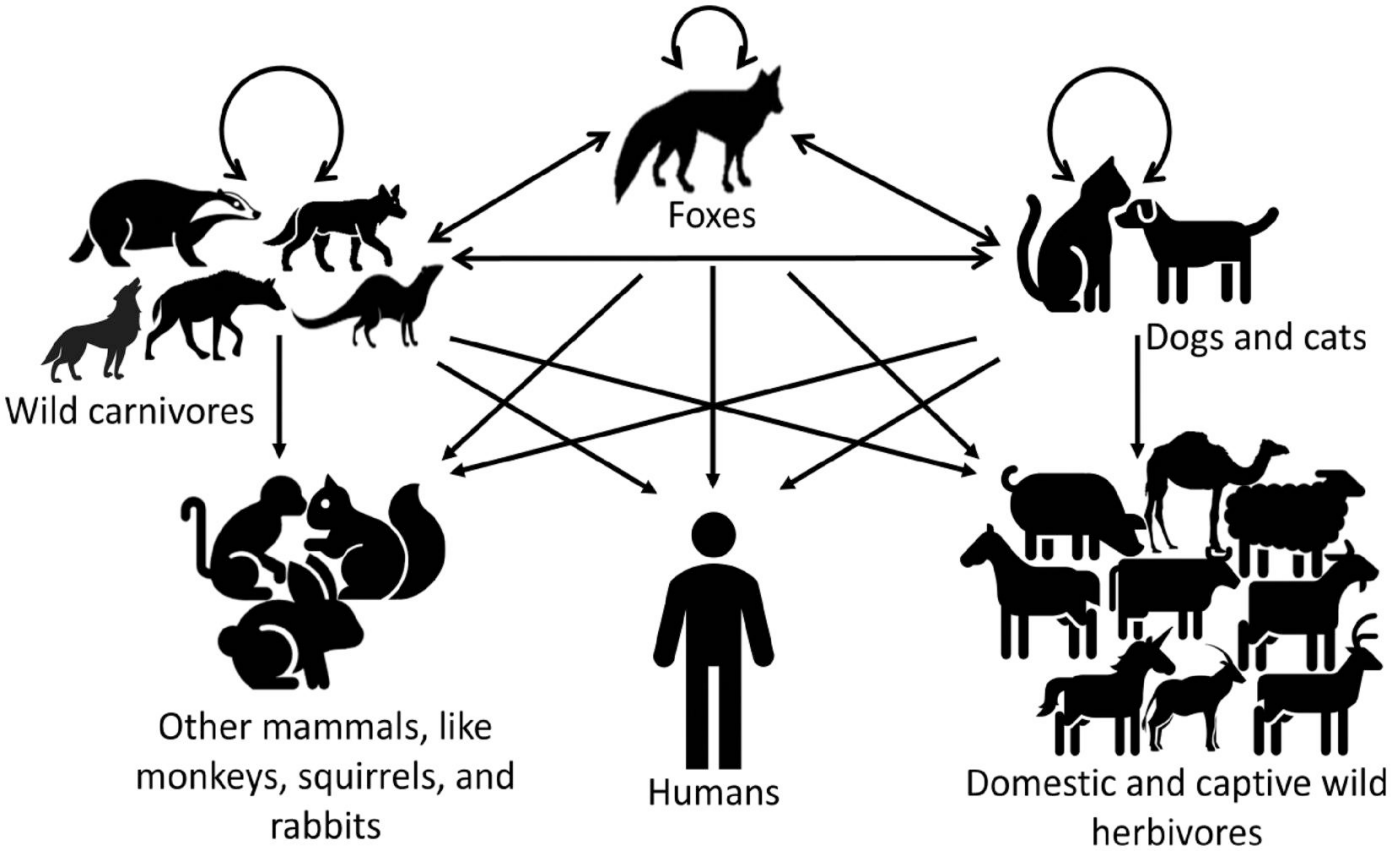
Possible transmission dynamics of rabies at the human–animal interface in the Arabian Peninsula.

The review revealed that foxes are the primary rabies reservoir in the Arabian Peninsula. This virus persists naturally in foxes and other wild carnivores and is transmitted to humans, dogs, cats, captive wild and domestic animals, and other mammals (Fig. 4).

### Risk factors

#### High-risk locations

Figure 1 shows an estimate of the historical spatial and temporal distribution of rabies cases in the Arabian Peninsula. In Iraq, rabies was reported in almost all provinces except Dahuk and Arbil, with frequent reports in Babil, Baghdad, Wasit, Diyala, Basrah, and Salah al-Din, followed by Kirkuk, Al-Muthanna, and Maysan [19, 20]. Regularly reported areas in Jordan include Irbid, Mafraq, Amman, and Madaba [25]. In Oman, all provinces except Musandam have reported rabies [26], with Al Dakhiliyah, Al Batinah North, Al Batinah South, Ash Sharqiyah North, Ash Sharqiyah South, Muscat, and Dhofar showing a high number of cases [18]. Significant rabies locations in Qatar include Shahaniyah [22], Eastern and Qassim provinces in KSA [3, 4], and Al Ain and Dubai in the UAE [12]. In Yemen, key locations include Amanat Al Asimaha, Ibb, and Dhamar [27].

#### Animal bites

Dog bites are the most common in the region, followed by bites from other animals, such as foxes, wolves, and camels. However, carnivore bites or scratches carry a higher rabies risk [15, 17, 18]. Any animal showing neurological dysfunction should be considered a potential rabies threat. Bites are mostly reported on the upper and lower extremities, followed by the body, head, and neck [16, 18].

#### Organ transplantation

Organ transplantation has been identified as a risk for rabies transmission in the region, as two recipients in Kuwait and KSA suspected of contracting the infection from an Indian donor [28].

#### Human age, sex, and occupation

Adolescents often provoke animals and may lack awareness of how to prevent dog bites [9]. Men, especially the young, are more likely to encounter rabid animals [16, 18, 20, 27]. Animal caregivers face a higher rabies transmission risk due to exposure to scratches, bites, or saliva from rabid animals.

#### Knowledge and attitude regarding rabies

Knowledge and attitudes significantly influence rabies prevalence. Following an animal bite, prompt first aid, observing the animal, and post exposure vaccination are crucial. Neglecting these measures increases the risk, as observed in KSA and Oman [29].

#### Vaccination and its coverage

Rabies preexposure prophylaxis (PrEP) is crucial for pet carnivores in the prevention of rabies, while postexposure prophylaxis (PEP) is essential for effective rabies protection. Although GCC countries mandate rabies vaccination, coverage is low (18.5%), increasing outbreak risk [9, 11]. PEP strategies exist across the region, but availability can vary, as seen in Oman in 1997 when vaccine shortages led to rabies cases [29].

#### Stray animals

Stray animals are widespread and gradually increasing in the region [13], heightening the risk of rabies. However, Qatar is addressing this issue by trapping and sheltering stray dogs [12].

#### Risks related to livestock and farm animals

Livestock and farm animals are at risk of attack by stray and wild animals in the desert farming system, as seen in Qatar [22]. Poor monitoring and evaluation, along with limited PEP, heighten this threat [30].

#### Residence

Rural areas pose a higher rabies risk [21] due to inadequate healthcare facilities, negligence, lack of knowledge, and a higher presence of stray dogs and wild carnivores.

#### Season

Open or unsafe camping, common cultural practice in the desert during the winter, increases the risk of wild carnivore attacks, potentially spreading rabies, as seen in Oman [29].

#### War and famine

Civil unrest results in an increase in exiled and stray animals, intensifying human–wildlife interactions. Conflict-affected nations, grappling with financial strain and compromised health systems, struggle to implement regular rabies control measures, as seen in Iraq and Yemen [14].

#### Animal ownership

Pet ownership can elevate the rabies prevalence, if there is inadequate animal care and vaccination [31], as contact with stray and wild animals heightens the risk.

### The expert opinion

Experts agree on the significant public health impact of rabies in the Arabian Peninsula but express scepticism about the reliability of published data on rabies cases. They believe that, in addition to regular reports, many cases go unreported. Insufficient details on reported cases, surveillance systems, tested animals, sampling methods, and representativeness pose challenges for decision-making in rabies control. Emphasizing robust diagnostic surveillance, experts stress the importance of confirming the presence or absence of rabies in a country and advocated for molecular assessment to identify reservoirs, particularly regarding fox species. Clarity in reservoir identification is also crucial before proceeding with wildlife vaccination. Concerns about the cost of wildlife vaccination prompted suggestions for risk assessments to guide effective interventions. Effective management of stray and free-roaming dogs is essential for controlling rabies, focusing on gathering data on bite incidents, ensuring accessibility to PEP, and monitoring the neurological outcomes of affected individuals.

Regional cooperation among the countries of the Arabian Peninsula was recommended to prevent and control rabies collectively and addressing cross-border virus spread. Despite reporting gaps and delays, experts highlighted the importance of assessing rabies burden and investing in surveillance and mass dog vaccination to confidently declare areas rabies-free. A comprehensive, collaborative, and data-driven approach to rabies control is urged, focusing on improving the reliability of prevalence data, accurately identifying reservoir animals, conducting risk assessments, and fostering regional cooperation to address cross-border disease spread.

## Discussion

The global goal is to eliminate human deaths from dog-mediated rabies by 2030. However, particular emphasis is needed in the Arabian Peninsula to eliminate human rabies caused by foxes. Achieving zero human deaths requires an integrated One Health approach to control rabies transmission at the human–animal interface through the implementation of the following activities:

### Surveillance

Multi-sectoral surveillance is pivotal for rabies prevention and control. It ensures the management of rabies cases, monitoring of interventions, handling of animal bite cases, and estimation of disease burden. Surveillance is critical in endemic countries like Oman, Yemen, Jordan, and Iraq. It is also essential in countries such as Qatar, Kuwait, and KSA, where sporadic rabies cases occur. Surveillance should include systematic sample collection, investigation of sudden neurological signs in humans and animals, laboratory analysis, and data interpretation [32].

### Human prophylaxis

The safety, efficacy, and administration of rabies vaccines and rabies immunoglobulins (RIG) are well-established. Rabies vaccines are believed to provide lifetime immunity, remaining effective even if neutralizing antibody titres decline [33]. The rabies vaccine is recommended for both PrEP and PEP, while RIG is highly recommended for PEP [34]. Administration strategies and dosage depend on the intended use, adhering to guidelines from producers, the CDC, WHO, and local recommendations [35].

Several licensed inactivated virus vaccines are available for rabies, including human diploid cell vaccine (HDCV; Imovax Rabies and Rabivac), purified chick embryo cell vaccine (PCECV; RabAvert and Rabipur), purified vero cell rabies vaccine (PVRV; Verorab, Imovax-Rabies Vero, Rabivax-S, TRC Verorab), and purified duck embryo vaccine (Lyssavac N). PVRV and PCECV are commonly used in resource-limited countries due to their cost-effectiveness and similar safety and effectiveness to HDCV [36]. Rabies messenger RNA vaccines are currently being studied and have shown promise in early clinical trials [37].

Establishing an effective rabies vaccination strategy is of utmost importance in this region. This involves developing a comprehensive national strategic plan with dedicated focal points and committees for overseeing vaccination preparation, implementation, monitoring, and evaluation. Individuals handling the virus or rabid animals are at high risk and should receive PrEP. PEP should be administered upon confirming rabies exposure [32]. If previously vaccinated individuals are exposed to rabies, a shortened course of PEP is recommended [35].

RIG, obtained from hyperimmunized human or equine donors, is a crucial component of the PEP regimen for rabies [38]. The recommended dose of hyperimmunized immunoglobulin is 20 international units per kilogram of body weight for individuals of all ages. Although PEP failures are extremely rare, deviations from the protocol, such as delays or improper administration, can lead to fatal outcomes [39]. It is noted that the PEP strategy is implemented across the region, however, affordability challenges in countries like Yemen result in several human rabies cases each year.

### Canine vaccination

Mass canine vaccination, using killed or attenuated vaccines, is essential for wild and domestic carnivores in Oman, KSA, Iraq, and Jordan. However, success relies on a thorough understanding of local carnivore population and ecology. To maintain necessary herd immunity, it is recommended to schedule one to two vaccination campaigns annually, achieving at least 70% coverage. While parental vaccination remains the primary method, ORV has successfully controlled rabies, especially in wildlife and difficult-to-restrain stray dogs. ORV is vital for preventing disease spread and eliminating rabies when wildlife is the primary host [40]. Recombinant rabies vaccines are available for veterinary use [1], but further research is necessary to compare their effectiveness with existing vaccines.

A successful ORV strategy necessitates consideration of species-specific factors, including total population, local ecosystem, rabies transmission dynamics, baiting methods, spatial distribution, timing, frequency, and bait abundance. However, ORV may not consistently achieve high seroconversion rates due to challenges in delivery and immunology. This method relies on carnivores being attracted to the bait, consuming it, and properly depositing the vaccine onto their oral mucosa. As an alternative to ORV, a ‘trap-vaccinate-release’ approach using the parenteral route may be considered [32].

### Carnivore population management

Eliminating rabies in this region requires addressing all carnivores, with population management through birth control, particularly sterilization, when conditions such as high vaccination coverage, cost-effectiveness, and ecological requirements are met. However, killing or depopulating carnivores is not recommended [32].

### Control of cross-border movement

The international movement of animals poses a public health risk, facilitating the potential introduction, emergence, or re-emergence of rabies in new areas like Qatar, Bahrain, and Kuwait. Import regulations for mammals must adhere to WOAH standards, requiring a valid international veterinary certificate of rabies vaccination [32].

### Community education and practice

Successful vaccination and canine population management campaigns depend on community education, awareness, engagement, and mobilization. In endemic areas, educating children about safe animal bite care is crucial. Public awareness is essential, particularly in emphasizing the high risk of rabies transmission from slaughtering or consuming rabid or suspected animals [32].

### Political willingness

Accurate and current data are pivotal for a comprehensive understanding and developing effective intervention strategies for rabies. Unfortunately, there is a noticeable dearth of data on rabies in countries in the region. Publishing governmental rabies records or reporting to WHO and WOAH may present sensitivities and require cross-ministerial approval, necessitating political commitment. Reporting human and animal rabies data as well as information on animal bites is critical for the WHO and WOAH. Without baseline data, it is challenging to declare a country free of rabies definitively.

### Multidisciplinary approach

Effectively controlling rabies at the human–animal interface necessitates a multidisciplinary team. Standardized procedures for rabies surveillance and outbreak investigations, adherence to recommended diagnostic protocols, and collaboration with national public health and animal health departments, as well as international organizations, like WOAH and WHO are essential [32].

## Conclusion

This comprehensive review and expert opinion analyse the historical rabies landscape in the Arabian Peninsula. Though rabies is prevalent in Iraq, Jordan, Oman, and Yemen, documented cases suggest significant underreporting. The findings emphasize the urgent need for One Health surveillance using the SARE concept in the region, aiming for zero dog-related deaths by 2030 and improved public health outcomes. The Gulf-CDC and its partners should initiate a targeted project on risk assessment and rabies transmission dynamics at the human–animal interface, particularly focusing on the human–fox interface.

## Supplementary Material

ckae164_Supplementary_Data

## Data Availability

All data were derived from publicly available sources and are included in the online supplementary material.
Key points Significant underreporting of rabies cases in the Arabian Peninsula limits the effectiveness of current control efforts. There is scepticism among experts regarding the accuracy of rabies reports in journals. To address rabies in the region, it is essential to adopt a One Health approach that incorporates the Stepwise Approach towards Rabies Elimination. Implementing robust surveillance systems and targeted public health interventions is necessary to achieve the 2030 goal of zero dog-mediated human rabies deaths. Strengthening cooperation among Gulf-CDC and regional partners will facilitate risk assessment and targeted strategies at the human–animal interface, across the Arabian Peninsula.
