# Supplementary material for: Epidemiology, transmission dynamics, risk factors, and future directions of rabies in the Arabian Peninsula using one health approach: a review
Source: Eur J Public Health. 2025 Jan 13;35(Suppl 1):i14–22. doi: 10.1093/eurpub/ckae164 (PMC11725953; doi:10.1093/eurpub/ckae164)
Supplement: ckae164_Supplementary_Data [file ckae164_supplementary_data.zip › ckae164_Supplementary_Data/ejph-2024-02--0115-File006.pdf]

Epidemiology, Transmission Dynamics, Risk Factors and Future Directions of Rabies in the Arabian Peninsula Using One Health Approach: A review

Supplementary Table 1: Baseline data extracted from the selected articles

Sl. No.	Author, country, and article type	Animal cases	Human cases
1	Abdulmoghni et al., 2021 (1)<br>Yemen; Original article *	2011-2017<br>Total: 76049/21927 (confirmed as suspected and confirmed)	2011-2017<br>Total exposure (animal bite)/ positive exposure/death (confirmed)<br>Total: 76049/21927/295<br>Exposure<br>Male: female = 50882: 25167<br>0-4 years: 10647/3289/30<br>5—14 years: 31941/8990/143<br>15-40 years: 20533/6140/77<br>>40 years: 12928/3508/44<br><br>Rabies cases<br>Cases: 2011: 38; 2012: 49; 2013: 44; 2014: 43; 2015: 40; 2016: 32; 2017: 49
2	Abdulmoghni et al., 2021 (2)<br>Yemen; Abstract	2011-2018<br>Total: 89590/25574	2011-2018<br>Total exposure (animal bite)/ positive exposure/death (confirmed): 89590/25574/347
3	Ahmed et al., 2020 (3)<br>Oman; Original article*	Camel (2009-2013)<br>Method: dFAT, RT-PCR, sequencing of N gene nucleotide, histopathology<br>Camel: 45/22<br>Histopathology found perivascular lymphocytic and monocytic cuffs in the brain. Negribodies were identified in cerebrum hippocampal pyramidal neurons. Some (n=20) dFAT positive samples showed neuronal necrosis, neurophagia babe's nodule, and neuronal degeneration and necrosis. Neuronal cell swelling, cytoplasmic vacuolation, and gliosis were also reported. In addition, edema, diffuse hemorrhage, satellitosis, meningitis, and malacia were observed.<br>Two sequences: GU35316 and KC883998	
4	Al Abaidani et al., 2015 (4)<br>Oman; Original article*	2006-2013<br>Animal rabies cases: 758/425<br>Fox: 61/47; Dog: 7/2; Cat: 8/0; Camel: 67/40; Sheep/Goat: 546/312; Other: 66/22;<br><br>Al Dhahira: 44/17; Al Buraimi: 1/1; Al Batinah North: 166/89; Al Batinah South: 17/10; Al Dakhiliyah: 342/201; Ash Sharqiyah North: 32/22; As Sharqiyah South: 99/52; Dhofar: 39/26; Muscat: 18/7	1991-2013<br>Animal bites: 22778<br>Gender: Male: 15938; Female: 6784<br>Age: Major cases were in the 10-19 years age group<br>Region: Majority of the cases were from Muscat<br>Biting location: Upper extremities (hands) (11143), lower extremities (8067), body (1003), head and face (1595); others (980)<br>Cat: 11002, Dogs: 8021; Fox: 1185; Wolf: 46; Others: 1276<br>Incidence of animal bites increased from 1991 to 1999, then gradually decreased after 2005.<br><br>Human rabies cases: 8<br>1990: 1; 1991: 1; 1992: 1; 1993: 1; 1997: 1; 1998: 1; 1999: 1; 2003: 1

Sl. No.	Author, country, and article type	Animal cases	Human cases
			Male: 3; Female: 5; Omani: 7; Bangladeshi: 1<br>Biting animals: Fox: 5, Wolf: 1; Dog: 1; Wild cat: 1<br>Locations Dhahira: 1; Dhofar: 4; North Batinah: 1; North Sharqiyah: 1; Buraimi: 1
5	Al-Eitan et al., 2021 (5)<br>Jordan; Original article*	Study year: 2019<br>Dog: 7/5; Cow: 2/0; Fox: 2/2; Sheep: 1/0; Total: 12/7 animals<br>Methods: FAT, RT-PCR, Whole genome sequencing<br>Area: Balqa: 4/3; Madaba: 1/1; Amman: 1/1; Ibrid: 4/1; Tafilah: 1/1; Zarqa: 1/0	
6	Al-Habsi et al., 2011 (6)<br>Oman; Case report*	First case of dog rabies in Oman<br>Adult stray dog in the Musanaa district<br>FAT positive	
	Al-Hadithi et al., 2008 (7)*<br>Yemen	Total: 1695/1233<br>Carnivores: 1626/1177; Cats: 28/15; Dogs: 1590/1155; Foxes: 8/7; Herbivores: 43/36; Omnivores: 26/20; Cattle: 21/17; Donkeys: 18/15; Sheep: 4/4	
7	Al-Qudah et al., 1997 (8)<br>Jordan; Original article*	1982-1995<br>Total cases: 419/164<br>Stray dogs: 74; Cows: 32; Pet dogs: 14; Foxes: 13; Wolves: 5; Donkeys: 8; Camels: 4; Sheep/Goats: 6; Stray cats: 2; Pet cats: 2; Hyenas: 1; Pet monkey: 1; Badgers: 2<br>Cases by year: 1982: 12; 1983: 21; 1984: 4; 1985: 12; 1986: 5; 1987: 5; 1988: 6; 1989: 6; 1990: 9; 1991: 7; 1992: 24; 1993: 14; 1994: 23; 1995: 16<br>Cases by city: Irbid: 48; Mafrak: 27; Amman: 35; Madaba: 18; Zarka: 9; Maan: 7; Ramtha: 6; Tfileh: 4; Balqa: 3; Aqaba: 1; Jordan Valley: 6	5 human cases from dog bites
8	Al-Rawashdeh et al., 2000 (9)<br>Jordan; Original article*	April 1996-February 1998<br>Camels: 369/8	
9	Al-Shamahy et al., 2013 (10)<br>Yemen; Original article*	2011<br>Total: 180/166<br>Dogs: 166/104; Foxes: 3/3; Donkeys: 3/3; Cats: 3/1; Goats: 2/2; Hyenas: 1/1; Cows: 1/0; Rats: 1/0<br>Male animals: 127/77; Female: 53/37; Owned animals-: 106/59; Stray animals: 74/55; Rural: 156/100; Urban: 24/14	Human cases:<br>Total exposure (animal bite)/ positive exposure: 180/166<br>Animal attacked individuals: Males: 124/78; Females: 56/36; ≤10 years: 101/74; 11-20 years: 47/26; >20 years: 32/14<br>Biting sites: Upper extremities: 79/53; Head and neck: 34/26; Lower extremities: 67/35
10	Alknawy et al., 2018 (11)<br>Saudi Arabia; Case report		2016<br>First human rabies case in Saudi Arabia involved a 60 year old Saudi man; imported from Morocco; Dog scratched his face
11	Ata et al., 1993 (12)<br>Oman; report*	First animal case in 1990; two foxes and one goat<br><br>1990-1991	The first human case of rabies occurred in 1990; involving a school boy from the Dhahira

Sl. No.	Author, country, and article type	Animal cases	Human cases
		Total: 203/127<br>Fox: 165/99; Dog: 3/2; Cat: 9/6; Camel: 3/2; Cattle: 7/7; Goat: 9/6; Others: 7/5<br>Al Dhahirah: 7/6; Al Buraimi: 1/1; Al Batinah North: 20/18; Al Batinah South: 45/31; A'Dakhiliya: 49/31; Samail: 9/8; A'Sharqiyah: 41/23; Salalah: 31/9	region who was attacked by a fox and contracted the infection<br>The second case was reported in 1991, in Jiddat al Harashis (Haima)
12	Awaidy and Al Hashami 2020 (13) Oman; review		The first human case was reported in 1990, involving an Omani male from Dhakira governorate. A second case was recorded in 2003<br>Oman launched rabies surveillance in 1990<br>Imported cases were reported at an average of one case per year<br>The last case occurred in 2016<br>Oman is considered endemic for rabies
13	Bales Jr et al., 1982 (14)<br>Saudi Arabia; Review	The first human case in the Eastern Province of Saudi Arabia was reported in 1982. The first documented rabies case was in 1974 in a horse, that had been bitten by a fox in Qaisumah.<br>In 1977, a dog near Dhahran was affected, followed by another fox in Shedgum, and another fox in Ain Nakhl in 1978. A dog was reported in 1981 near Dhahran.	
14	Body et al., 2014 (15)<br>Oman; Original article*	2011-2012<br>Different animal species: 257/135<br>Method: FAT, RT-PCR, sequencing<br>Governorate: Ad Dakhliyah and Ash Sharqiyah<br>Four sequences: Goat: KC883999 and KC884000<br>Camel: KC883998; Sheep: KV883997	
15	Carrara et al., 2013 (16) Review Yemen	Two rabies cases were either imported or exported from 1980 to 2012:<br>1991: Yemen to Russia<br>2009: India to UAE	
16	Dhayhi et al., 2019 (17)<br>Saudi Arabia; Case report		The first human case in Saudi Arabia involved a Yemeni refugee, a young boy in 2016, who was bitten by a stray dog in the Jazan region.
17	El-Neweshy et al., 2020 (18)<br>Oman; Original article*	2017-2019<br>Animals: 117/64<br>Fox: 8/7; Dog: 2/0; Cat: 1/0; Bat: 3/0; Camel: 14/12; Cattle: 7/4; Sheep: 8/3; Goat: 70/34; Gazelle: 3/3; Oryx: 1/1<br>Regional description: Al Dakhiliyah: 30/14; Al Dhahirah: 12/7; Al Batinah North: 16/8; Al Batinah South: 5/2; Al Buraymi: 1/0; Al Wusta: 8/8; As-Sharqiyah North: 6/2; As Shrqiyah South: 27/18; Dhofar: 3/3; Muscat: 9/2	
18	Fagbo et al., 2021 (19, 20)<br>KSA; Case report and conference abstract*	2011<br>Qassim region<br>Sand cat case: 1/1	
19	Faizee et al., 2012 (21)<br>Jordan; Original article*	July 2009- May 2010<br>Total: 29/27<br>Dog: 13/11; Cat: 1/1; Fox: 1/1; Pig: 1/1; Cow: 5/5; Sheep: 2/2; Goat: 1/1; Camel: 1/1; Horse: 1/1; Donkey: 3/3.	

Sl. No.	Author, country, and article type	Animal cases	Human cases
		Methods: FAT, Histopathology; Immunohistochemistry; RT-PCR	
20	Gautret et al., 2021 (22)<br>Letter<br>UAE<br>Qatar		Human rabies imported cases:<br>2009: UAE from India<br>2014: Qatar from India<br>2015: Kuwait from India<br>2016: Saudi Arabia from Morocco<br>2016: Oman from Bangladesh<br>2018: Qatar from Nepal<br>2019: Qatar from Nepal<br>*All cases involved migrant workers, except the Saudi-Morocco case, which involved a traveler.*
21	Hananeh et al., 2015 (23)<br>Jordan;<br>Original article*	Total:11/10<br>Cow: 5/4, Donkey: 3/3, Goat: 2/2, Horse: 1/1	
22	Horton et al., 2013 (24)<br>Iraq;<br>Original article*	2001-2010<br>Animal: 40/3; Dogs: 38/1; Cattle: 2/2<br>Sequences: Cattle: JX524176, JX524178; Dog: JX524177	Total: 186 cases<br>Male: 166; Female: 20; Urban: 32; Rural: 154; 0-14 years: 117; 15+: 69
23	Horton et al., 2015 (25)<br>Middle East;<br>Original article	*Viruses from the Middle East occur across three of the four clades. Viruses from Jordan belong to clade B and appear to be introductions from Iran and the Arabian Peninsula, largely derived from wildlife, with evidence of spill over into dogs. In contrast, there is evidence that in Iraq, viruses from this wildlife associated clade B and the dog associated clade D are co-circulating. Data from the Arabian Peninsula including Saudi Arabia, United Arab Emirates, and Oman support a single, relatively recent incursion into the Arabian Peninsula*<br>*region within clade B, which is descended from a common ancestor that likely existed in the Arabian Peninsula (location probability 92%) approximately 40 years ago. This suggests the endemic maintenance of infection in a local reservoir for the past 40 years, with the majority originating from wildlife.*	
24	Hussain et al., 2013 (26)<br>Oman; Original article*	2006-2010<br>Total: 444/279<br>Al Batinah: 54/38; Dakhiliyah: 250/154; A'Dhahira: 22/16; Ash Sharqiyah: 86/50; Dhafor: 27/16; Muscat: 5/5<br>Cattle: 49/14; Sheep: 72/40; Goat: 226/158; Camel: 39/29; Fox: 50/37; Dog: 5/1; Cat: 1/0; Bat: 1/0; Deer: 1/0	
25	Ismail et al., 2020 (27)<br>Iraq; Original article*	2013-2017<br>Total cases: 200+58=258/53+19=72; Cattle: 38; Dogs: 15+19=34<br>2013: 17; 2014: 19; 2015: 4; 2016: 4; 2017: 9	2012-2017<br>Animal bites: 2012-2016: 12358<br>Total cases: 54<br>2013-2017: Male: 47; Female: 5, 0-15 years: 38, 15+: 14
26	Kasem et al., 2019 (28)<br>Saudi Arabia;<br>Original article*	2010-2017<br>Total: 199/158<br>Dog: 47/3; Cat: 19/13; Fox: 24/18; Wolf: 6/4; Sheep: 31/26; Goat: 29/26; Camel: 40/34; Procavia capensis: 1/1; Monkey: 2/2<br>Region wise<br>Riyadh: 29/25; Al-Qassim: 84/63; Eastern region: 59/48; Northern boundaries: 1/1; Jouf: 3/3; Al-Madina: 14/10; Hail: 4/3; Gazan: 2/2; Tabuk: 3/3	
27	Memish et al., 2015 (29)<br>Saudi Arabia;<br>Short communication*	2007-2009<br>Animal rabies<br>Total: 40/37;<br>Camel: 5/5; Cow: 2/2; Dog: 11/11; Fox: 9/6; Goat: 4/4; Sheep: 6/6; Wolf: 3/3	Animal bites: 11,069<br><br>Temporal: 2007: 3153; 2008: 4333; 2009: 3583

Sl. No.	Author, country, and article type	Animal cases	Human cases
		Temporal: 2005: 8/8; 2007: 10/10; 2008: 9/6; 2009: 10/10; 2010: 3/3	Species: Fox: 142; Wolf: 58; Dog: 5482; Cat: 3276; Monkey: 72; Rat: 529; Mouse: 868; Camel: 351; Other: 291
28	Novelli and Malankar, 1991 (30)<br>Oman; Short communication*	1991<br>Fox: 2/2	April 1990 - October 1990<br>Total (fox bites): 46/1<br><br>In 1990 (between April and October), 46 fox bite cases were recorded, most of which involved males (65%) and children (28%).
29	Oude Munnink et al., 2020 (31)<br>Qatar; Case report*	2018<br>Animals: 2; local fox and camel AlKharsha	2018<br>Humans: 2, imported from Nepal
30	Rollin et al., 1985 (32)<br>Kuwait; Review	1981: Fox: 1<br>1982: Sheep: 1<br>1984: Fox: 1	
31	Saeed and Al-Mousawi, 2017 (33)<br>Kuwait; Case report		Transmission through organ transplantation<br>Human: One five-year-old girl received a kidney from a donor; who was suffering from rabies<br><br>*Another kidney recipient died. Two other patients, one who received a liver and another who received a heart, also died. Two additional patients who received corneas were asymptomatic and took post-exposure prophylaxis (PEP).*
32	Scrimgeour and Mehta, 2001 (34)<br>Oman; case report		1997: A woman was bitten by a fox.<br>*1990: The first case of human rabies in Oman was reported:. a child in Buraimi, northern Oman, was bitten by a fox and died.*<br>*1991: a 45 year old woman was bitten by a wolf (12).*<br>*1992: a five month old female child was bitten by a fox (12).*<br>*Additionally, in 1999, a 13 year old girl was scratched by an unidentified animal, suspected to be a feral cat.*
33	Scrimgeour et al., 1999 (35)<br>Oman;<br>Review	*After the first human case in 1990, further human cases occurred in 1991 (wolf bite), 1992 (fox bite), and 1997 (fox bite). From 1990 to1997, 1,195 bites were reported, with 45% attributed to dog bites and 4% to fox bites. Animal rabies was reported in 163 foxes, 25 cattle, 11b camels, and 11 dogs. In 1997, 11 foxes tested positive for rabies.*	
34	Seimenis, 2008 (36); Middle East; Review	WHO report: Jordan<br>1988: 65 reported cases in domestic animals<br>2005-2006: 8 cases in domestic animals<br>1996: 4 dogs and 1 cat<br><br>*Foxes and Jackals are the most important vector species for rabies in the Middle East. Additionally, a few other species, such as badgers, mongooses, and grey wolves are also epidemiologically significant.*	WHO report:<br>Iraq: 39 cases from 2005-2006<br>Jordan: 6 cases from 1992-1996, 0 cases from 1997-2002, and 0 cases from 2003-2006<br>Kuwait: 0 cases from 1992-1994 and 1996-2004, with 1 case in 1995<br>Oman: 1990-1993: 3 cases; 1994-1996: 0 cases, 1997-1999: 3 cases; 2001-2004: 1 case<br>Qatar: 1988-1999: 0 cases<br>Saudi Arabi:<br>1985-1995: 43 cases, 1996-2000: 6 cases, 2005-2006: 7 cases

Sl. No.	Author, country, and article type	Animal cases	Human cases
			Yemen<br>1990-1995: 72 human cases; 1997: 20 human cases; 1998-2000: 0 cases
35	Stanley, 1990 (37) Yemen; Short communication*	1982-1986<br>Total: 1101/591<br>Temporal: 1982: 10/4, 1983: 10/69, 1984: 265/150, 1985: 358/177, 1986: 368/191<br>Dog: 570, Cat: 4, Cow: 6, Donkey: 3, Goat: 2, Fox: 5, Hyena: 1	
36	Taylor et al., 2021 (38): Review		*In 2017 in Iraq*<br>*Human cases: 9*<br>*Canine cases: 8*<br>*Human exposure: 14725*
37	Wernery and Kumar, 1993 (39) UAE; Review	1991-1992<br>First rabies case in the UAE: 1991.<br>Suspected to have been brought from Oman<br>Goat: 4; Sheep: 6; Fox: 3; Camel: 31; Total: 44<br>Spatial: Al Ain: 24; Abu Dhabi: 2; UAQ: 2; Dubai: 16:<br>Total: 44	
38	WHO, 1992a and WHO, 1992b (40, 41)<br>Oman and UAE; Report and review*	UAE<br>17 cases of lab confirmed rabies were reported from October 1990 to July 1991 in Abu Dhabi and Umm al Qaiwain; Camel: 9; Sheep/Goats: 5; and Fox: 3<br><br>Oman<br>From 1990-1991: 185/123<br>Total animal cases: 185/120<br>Fox: 150/99; Cattle: 5/5; Goat: 16/10; Sheep: 1/1; Camel: 5/2; Cats: 2/1; Dogs: 2/2; Mongoose: 2/1; Jackals: 1/1 Others: 1/1	Oman:<br>2 cases involved in expatriate workers with origins outside Oman in 1983-1984
* Used in Meta-analysis			

Supplementary Table 2: Descriptive statistics of the reviewed articles

Characteristics	Number of articles, % (95%CI)	References
Study types		
Basic research	17, 41.46 (26.71-57.80)	(1, 3-5, 7-10, 15, 18, 21, 23-28)
Case report	10, 24.39 (12.91-40.64)	(6, 11, 12, 17, 19, 31, 33, 34, 40, 41)
Conference report/abstract	2, 4.88 (0.85-17.81)	(2, 20)
Letter to the editor	1, 2.44 (0.13-14.41)	(22)
Short communication	3, 7.32 (0.91-21.01)	(29, 30, 37)
Review	8, 19.51 (9.37-35.37)	(13, 14, 16, 32, 35, 36, 38, 39)
Publication year		
1981-1990	3, 7.32 (0.91-21.01)	(14, 32, 37)
1991-2000	8, 19.51 (9.37-35.37)	(8, 9, 12, 30, 35, 39-41)
2001-2010	3, 7.32 (0.91-21.01)	(7, 34, 36)
2011-2020	20, 48.78 (33.15-64.63)	(3, 4, 6, 10, 11, 13, 15-18, 21, 23-29, 31, 33)
2021-2024	7, 17.07 (7.70-32.65)	(1, 2, 5, 19, 20, 22, 38)
Host		
Human cases	21, 51.21 (35.36-66.85)	(1, 2, 4, 8, 10-13, 17, 22, 24, 27, 29-31, 33, 34, 36, 38, 40, 41)
Non-human cases	32, 78.05 (61.97-88.89)	(1-10, 12, 14-16, 18-21, 23, 24, 26-32, 36, 37, 39-41)

Supplementary Table 3: Spatial and temporal distribution of the molecular sequences of rabies virus along with host information reported in the Arabian Peninsula

Sl. No.	Country	Host	Sampling year (number of sequences)
1	Iraq	Cattle	Unknown (2), 2008 (6), 2010 (3)
		Unknown	Unknown (30)
		Dog	2004 (2), 2005 (2), 2007 (6), 2008 (7), 2009 (7), 2010 (2)
		Horse	2009 (2)
		Mongoose	2007 (2)
2	Jordan	Badger	1998 (1)
		Squirrel	1998 (1)
		Cattle	1998 (1)
		Donkey	1999 (1)
		Goat	1999 (1)
3	Oman	Goat	2003 (3), 2007 (2), 2009 (7), 2011 (1), 2012 (4)
		Cattle	2003 (3), 2004 (6), 2009 (2), 2012 (1)
		Sheep	2003 (3), 2009 (2), 2011 (1), 2012 (2)
		Fox	1990 (2), 1991 (2), 1998 (2), 2002 (6), 2009 (6), 2012 (1)
		Dog	2011 (1)
		Cat	2008 (3), 2009 (2)
		Camel	1991 (1), 2002 (3), 2004 (3), 2008 (1), 2009 (3), 2011 (1)
		Unknown	Unknown (3)
4	Qatar	Fox	2018 (1)
		Camel	2018 (1)
		Human	2018 (2)
4	Saudi Arabia	Fox	1987 (3), 1990 (1), 1997 (1), 1998 (1)
		Horse	1987 (1)
		Human	1981 (1)
5	United Arab Emirates	Fox	1991 (1), 2013 (1)
		Camel	1991 (3), 1992 (3), 1994 (2), 2013 (3)
		Dog	1994 (2)

References

1. Abdulmoghni RT, Al-Ward AH, Al-Moayed KA, Al-Amad MA, Khader YS. Incidence, Trend, and Mortality of Human Exposure to Rabies in Yemen, 2011-2017: Observational Study. JMIR public health and surveillance. 2021;7(6):e27623.
2. Abdulmoghni R, Almoayed K, Al Amad M. Patterns and distribution of human exposed to rabies in Yemen, 2011–2018. International Journal of Infectious Diseases. 2020;101:262.
3. Ahmed MS, Body MH, El-Neweshy MS, Alrawahi AH, Al-Abdawani M, Eltahir HA, et al. Molecular characterization and diagnostic investigations of rabies encephalitis in camels (Camelus dromedaries) in Oman: a retrospective study. Tropical animal health and production. 2020;52(4):2163-8.
4. Al Abaidani I, Al Abri S, Prakash KP, Hassan Hussain M, Hammad Hussain M, Al Rawahi AH. Epidemiology of rabies in Oman: A retrospective study (1991–2013). Eastern Mediterranean Health Journal. 2015;21(8):591-7.
5. Al-Eitan LN, Wu G, Golding M, Tang Y, Goharriz H, Marston DA, et al. Whole-genome sequencing and phylogenetic analysis of rabies viruses from Jordan. PLoS Neglected Tropical Diseases. 2021;15(5).
6. Al-Habsi SS, Al-Rawahi AH, Al-Far-e BH, Al-Kindi SA, Al-Lamki KA. Dog rabies: The first case reported from Sultanate of Oman. Asian Biomedicine. 2011;5(3):423-4.
7. AMK A-H, DYMA A-Q, RNA A. Epidemiological study of rabies in humans and animals in some governorates of the Republic of Yemen. Dhamar University Journal of Studies and Research. 2008;2008(9):27-53.
8. Al-Qudah KM, Al-Rawashdeh OF, Abdul-Majeed M, Al-Ani FK. An epidemiological investigation of rabies in Jordan. Acta Veterinaria. 1997;47(2-3):129-34.
9. Al-Rawashdeh OF, Al-Ani FK, Sharrif LA, Al-Qudah KM, Al-Hami Y, Frank N. A survey of camel (Camelus dromedarius) diseases in Jordan. Journal of zoo and wildlife medicine : official publication of the American Association of Zoo Veterinarians. 2000;31(3):335-8.
10. Al-Shamahy HA, Sunhope A, Al-Moyed KA. Prevalence of rabies in various species in Yemen and risk factors contributing to the spread of the disease. Sultan Qaboos University Medical Journal. 2013;13(3):404-10.
11. Alknawy M, Mohammed I, Ulla SN, Aboud AA. First confirmed case of human rabies in Saudi Arabia. IDCases. 2018;12:29-31.
12. Ata FA, Tageldin MH, al Sumry HS, al-Ismaily SI. Rabies in the Sultanate of Oman. The Veterinary record. 1993;132(3):68-9.
13. Awaidy SA, Al Hashami H. Zoonotic Diseases in Oman: Successes, Challenges, and Future Directions. Vector-Borne and Zoonotic Diseases. 2020;20(1):1-9.
14. Bales Jr JD, Choudury AA, Oertley RE. Rabies in Eastern Saudi Arabia. Saudi Medical Journal. 1982;3(3):195-201.
15. Body MHH, Rawahi AHA, Habsi SSA, Wadir AA, Saravanan N, Ahmed MS, et al., editors. Study on molecular characterization of rabies virus N gene segment from different animal species in the Sultanate of Oman2014.
16. Carrara P, Parola P, Brouqui P, Gautret P. Imported human rabies cases worldwide, 1990-2012. PLoS Negl Trop Dis. 2013;7(5):e2209.
17. Dhayhi NS, Arishi HM, Ibrahim AYA, Allah MBK, Hawas AM, Alqasmi H, et al. First confirmed case of local human rabies in Saudi Arabia. International Journal of Infectious Diseases. 2019;87:117-8.
18. El-Neweshy MS, Al Mayahi N, Al Mamari W, Al Rashdi Z, Al Mawly JH. Animal rabies situation in Sultanate of Oman (2017-2019). Tropical animal health and production. 2020;52(6):3069-76.
19. Fagbo SF, Al-Saigul AM, Ali AA, Elshamary E, Selim SA, Tatwany H, et al. Rabies in a Sand Cat (Felis margarita) in Saudi Arabia: One Health Implications. Journal of wildlife diseases. 2021;57(4):977-9.
20. Fagbo S, Asiri A. Sand cat Rabies in Saudi Arabia: Leveraging to Improve Spillover Surveillance and One Health Impact. International Journal of Infectious Diseases. 2022;116:S106-S7.
21. Faizee N, Hailat NQ, Ababneh MMK, Hananeh WM, Muhaidat A. Pathological, Immunological and Molecular Diagnosis of Rabies in Clinically Suspected Animals of Different Species Using Four Detection Techniques in Jordan. Transboundary and Emerging Diseases. 2012;59(2):154-64.
22. Gautret P, Al-Abri S, Al-Rawahi B, Memish ZA. Human rabies importation to the Middle East: An emerging threat? International Journal of Infectious Diseases. 2021;102:335-6.
23. Hananeh WM, Nassir IM, Ababneh MMK, Hailat NQ, Brown CC. Pathological and molecular diagnosis of rabies in clinically suspected food animals using different diagnostic tests. Large Animal Review. 2015;21(6):243-50.
24. Horton DL, Ismail MZ, Siryan ES, Wali ARA, Ab-dulla HE, Wise E, et al. Rabies in Iraq: Trends in Human Cases 2001-2010 and Characterisation of Animal Rabies Strains from Baghdad. PLoS Neglected Tropical Diseases. 2013;7(2).
25. Horton DL, McElhinney LM, Freuling CM, Marston DA, Banyard AC, Goharrriz H, et al. Complex epidemiology of a zoonotic disease in a culturally diverse region: phylogeography of rabies virus in the Middle East. PLoS Negl Trop Dis. 2015;9(3):e0003569.
26. Hussain MH, Ward MP, Body M, Al-Rawahi A, Wadir AA, Al-Habsi S, et al. Spatio-temporal pattern of sylvatic rabies in the Sultanate of Oman, 2006-2010. Preventive Veterinary Medicine. 2013;110(3-4):281-9.
27. Ismail MZ, Al-Hamdi NK, Al-Amery AN, Marston DA, McElhinney L, Taylor E, et al. Quantifying and mapping the burden of human and animal rabies in Iraq. PLoS Neglected Tropical Diseases. 2020;14(10):1-10.
28. Kasem S, Hussein R, Al-Doweriej A, Qasim I, Abu-Obeida A, Almulhim I, et al. Rabies among animals in Saudi Arabia. Journal of Infection and Public Health. 2019;12(3):445-7.

29. Memish ZA, Assiri AM, Gautret P. Rabies in Saudi Arabia: A need for epidemiological data. International Journal of Infectious Diseases. 2015;34:e99-e101.
30. Novelli VM, Malankar P. Epizootic of fox rabies in the Sultanate of Oman. Transactions of the Royal Society of Tropical Medicine and Hygiene. 1991;85(4):543.
31. Oude Munnink BB, Farag EABA, GeurtsvanKessel C, Schapendonk C, van der Linden A, Kohl R, et al. First molecular analysis of rabies virus in Qatar and clinical cases imported into Qatar, a case report. International Journal of Infectious Diseases. 2020;96:323-6.
32. Rollin PE, Sureau P, Atack WN. Rabies post-exposure prophylaxis in Kuwait. Trans R Soc Trop Med Hyg. 1985;79(2):267.
33. Saeed B, Al-Mousawi M. Rabies acquired through kidney transplantation in a child: A case report. Experimental and Clinical Transplantation. 2017;15(3):355-7.
34. Scrimgeour EM, Mehta FR. Rabies in Oman: Failed postexposure vaccination in a lactating woman bitten by a fox. International Journal of Infectious Diseases. 2001;5(3):160-2.
35. Scrimgeour EM, Mehta FR, Suleiman AJ. Infectious and tropical diseases in Oman: a review. Am J Trop Med Hyg. 1999;61(6):920-5.
36. Seimenis A. The rabies situation in the Middle East. Dev Biol (Basel). 2008;131:43-53.
37. Stanley MJ. Rabies in Yemen Arab Republic, 1982 to 1986. Tropical animal health and production. 1990;22(4):273-4.
38. Taylor E, Del Rio Vilas V, Scott T, Coetzer A, Prada JM, Alireza G, et al. Rabies in the Middle East, Eastern Europe, Central Asia and North Africa: Building evidence and delivering a regional approach to rabies elimination. Journal of Infection and Public Health. 2021;14(6):787-94.
39. Wernery U, B.N. K. Rabies in the U.A.E. Tribulus. 1993;3(1):15-21.
40. World Health Organization. Wildlife rabies in Oman and the United Arab Emirates. Relevé épidémiologique hebdomadaire / Section d'hygiène du Secrétariat de la Société des Nations = Weekly epidemiological record / Health Section of the Secretariat of the League of Nations. 1992;67(10):65-8.
41. World Health Organization. Rabies in Oman and the United Arab Emirates. The Lancet. 1992;339(8794):673.
