# Supplementary material for: Epidemiology, transmission dynamics, risk factors, and future directions of rabies in the Arabian Peninsula using one health approach: a review
Source: Eur J Public Health. 2025 Jan 13;35(Suppl 1):i14–22. doi: 10.1093/eurpub/ckae164 (PMC11725953; doi:10.1093/eurpub/ckae164)
Supplement: ckae164_Supplementary_Data [file ckae164_supplementary_data.zip › ckae164_Supplementary_Data/ejph-2024-02--0115-File007.pdf]

Epidemiology, Transmission Dynamics, Risk Factors and Future Directions of Rabies in the Arabian Peninsula Using One Health Approach: A review

Identification	Record search through four databases (Embase [*n* =245], PubMed [*n* =52], Scopus [*n* =99] and Web of Science [*n* =51]) resulted in a total of 447 records		
↓			
Screening	After removal of duplicates (*n*=269)	→	Papers removed (*n*=178)
↓			
Eligibility	Records assessed for eligibility after the removal of irrelevant articles (*n*=40)	→	Removed non-relevant papers like book chapters, reviews, articles in non-English languages, and non-rabies cases (*n*=229)
↓			
Included	Records assessed (*n*=49*)	←	Documents from grey literature and hand search (n = 9)

* Additionally, NCBI rabies genome records in the Arabian Peninsula were studied.

Supplementary Figure 1: PRISMA flow diagram illustration the systematic review process for selecting published articles on MERS-CoV in camels, including the criteria for inclusion and exclusion used in the study.

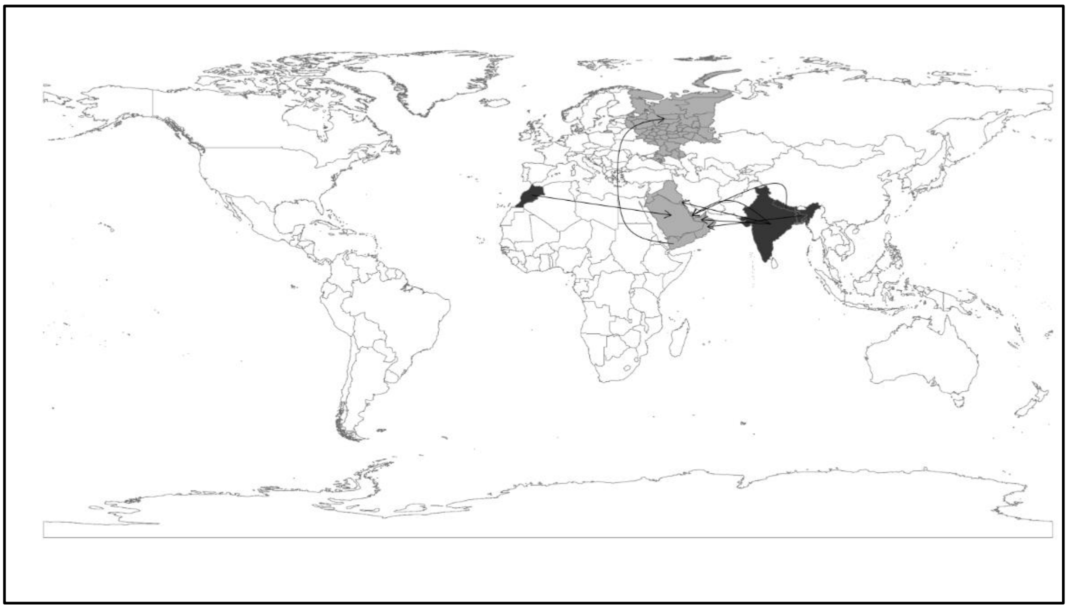

Supplementary Figure 2: Distribution of exported and imported human rabies cases from and to the Arabian Peninsula.

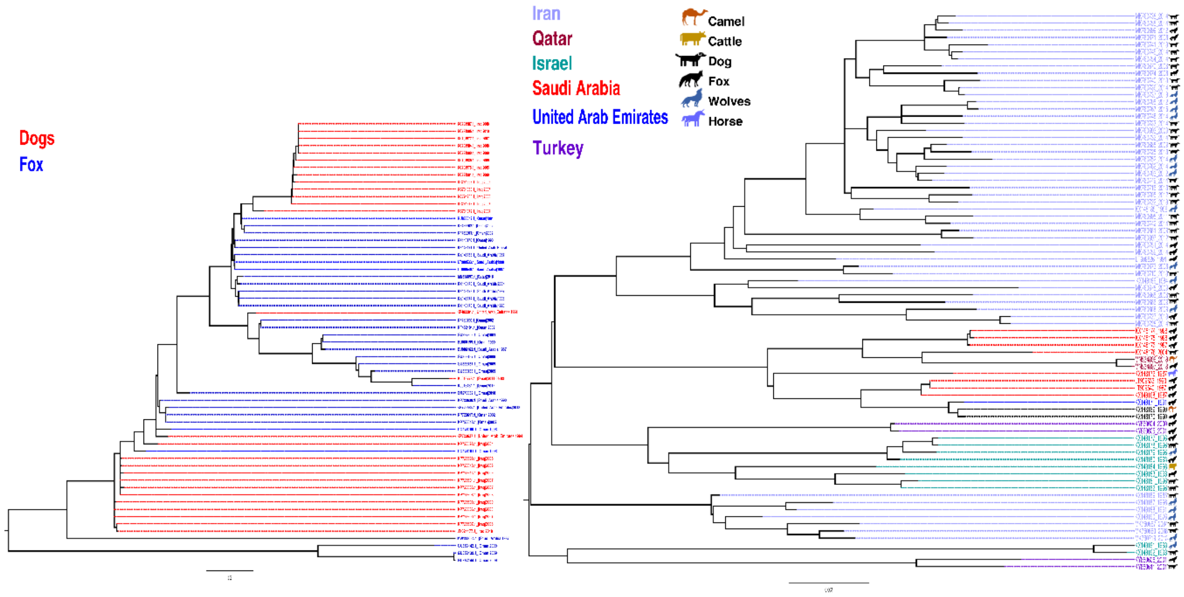

Supplementary Figure 3: Phylogenetic tree illustrating discrete phylogenetic reconstruction using only sequences from viruses detected in dogs or foxes in the Arabian Peninsula (Left panel); depicts the molecular relationships of the rabies virus top hits, showing BLAST results with 99-100% coverage and >95% identity of sequences isolated from various animals in the Arabian Peninsula (Right panel).
